# Supplementary material for: Identification of Sulfenylated Cysteines in Arabidopsis thaliana Proteins Using a Disulfide-Linked Peptide Reporter
Source: Front Plant Sci. 2020 Jul 2;11:777. doi: 10.3389/fpls.2020.00777 (PMC7343964; doi:10.3389/fpls.2020.00777)
Supplement: Supplementary file 1 [file Data_Sheet_1.zip › DATA/Sup Figure 1.DOCX]

Supplementary Material

## Supplementary Figure


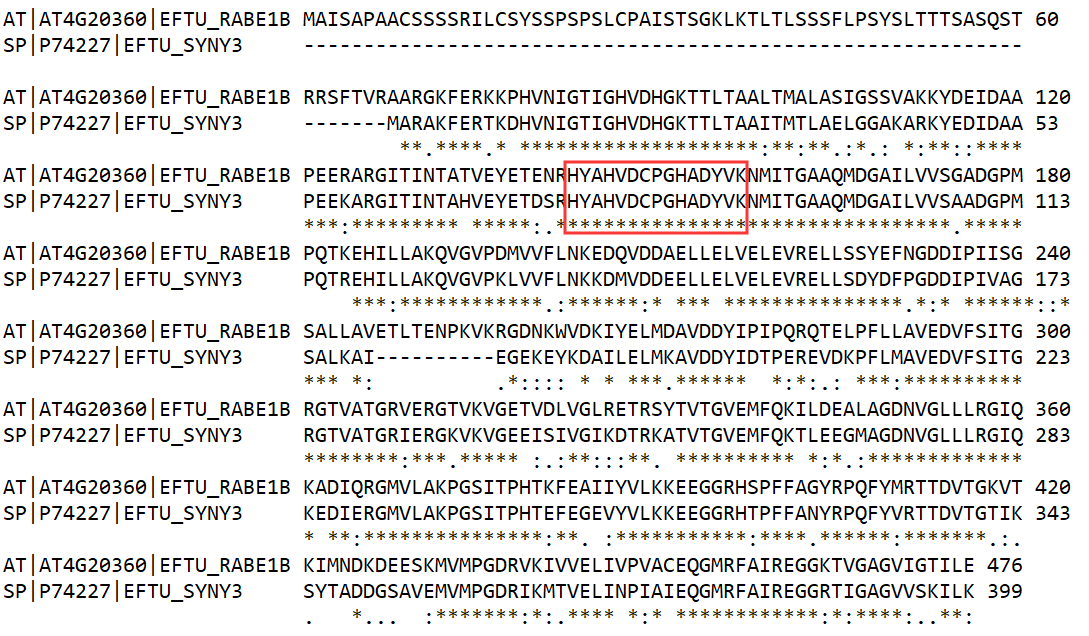


**Supplementary Figure S1 |** Protein alignment between EF-Tu proteins of *Cyanobacterium Synechocystis* (SP | P74227 | EFTU_SYNY3) and Arabidopsis (AT | AT4G20360 | EFTU_RABE1B). The peptide in red box was identified as YAP1C cross-linked in this study (**Supplementary Dataset S2**).
